# Transcriptional Coactivator TAZ Negatively Regulates Tumor Suppressor p53 Activity and Cellular Senescence

**DOI:** 10.3390/cells9010171

**Published:** 2020-01-09

**Authors:** Chiharu Miyajima, Yuki Kawarada, Yasumichi Inoue, Chiaki Suzuki, Kana Mitamura, Daisuke Morishita, Nobumichi Ohoka, Takeshi Imamura, Hidetoshi Hayashi

**Affiliations:** 1 Department of Cell Signaling, Graduate School of Pharmaceutical Sciences, Nagoya City University, Nagoya 467-8603, Japan; miyajima@phar.nagoya-cu.ac.jp (C.M.); y.kawarada@med.nagoya-u.ac.jp (Y.K.); pinkmonsoon1108@gmail.com (C.S.); mtkn218@gmail.com (K.M.); daisuke.b.m.1215@gmail.com (D.M.); 2 Department of Innovative Therapeutic Sciences, Cooperative Major in Nanopharmaceutical Sciences, Graduate School of Pharmaceutical Sciences, Nagoya City University, Nagoya 467-8603, Japan; 3 Division of Molecular Target and Gene Therapy Products, National Institute of Health Sciences, Kanagawa 210-9501, Japan; n-ohoka@nihs.go.jp; 4 Department of Molecular Medicine for Pathogenesis, Graduate School of Medicine, Ehime University, Ehime 791-0295, Japan; timamura.ind@gmail.com

**Keywords:** cellular senescence, oncogene, p300, p53, TAZ

## Abstract

Transcriptional coactivator with a PDZ-binding motif (TAZ) is one of the mammalian orthologs of *Drosophila* Yorkie, a transcriptional coactivator of the Hippo pathway. TAZ has been suggested to function as a regulator that modulates the expression of cell proliferation and anti-apoptotic genes in order to stimulate cell proliferation. TAZ has also been associated with a poor prognosis in several cancers, including breast cancer. However, the physiological role of TAZ in tumorigenesis remains unclear. We herein demonstrated that TAZ negatively regulated the activity of the tumor suppressor p53. The overexpression of TAZ down-regulated p53 transcriptional activity and its downstream gene expression. In contrast, TAZ knockdown up-regulated p21 expression induced by p53 activation. Regarding the underlying mechanism, TAZ inhibited the interaction between p53 and p300 and suppressed the p300-mediated acetylation of p53. Furthermore, TAZ knockdown induced cellular senescence in a p53-dependent manner. These results suggest that TAZ negatively regulates the tumor suppressor functions of p53 and attenuates p53-mediated cellular senescence.

## 1. Introduction

Transcriptional coactivator with a PDZ-binding motif (TAZ), also called WW domain-containing transcriptional regulator 1, has been identified as a 14-3-3 binding phosphoprotein [1]. TAZ is one of the mammalian orthologs of Yorkie, a transcriptional coactivator of the Hippo pathway of *Drosophila*. TAZ has been shown to stimulate transcription by interacting with a number of transcription factors [2]. TAZ plays an important role in the regulation of proliferation, differentiation, tissue growth, and organ morphogenesis [3,4]. TAZ activation has been widely observed in human tumors, in which TAZ was found to be essential for cancer development, progression, and metastasis [5]. Furthermore, many studies reported a correlation between elevated TAZ activation and the incidence of human cancer. Increasing evidence indicates that TAZ activation in cancer cells is oncogenic and also predicts a poor prognosis. A clearer understanding of the molecular mechanisms responsible for TAZ-mediated cancer progression will facilitate the development of the therapeutic targeting of TAZ.

The tumor suppressor p53 plays an important role in regulating cell proliferation during various stimuli, including genotoxic stress and oncogenic activation. The majority of cancers show abnormalities in the p53 pathway due to *TP53* mutations or the inhibition of p53 activation by other factors [6,7,8,9]. The most important function of p53 is to act as a transcription factor that activates various genes responsible for cell cycle arrest, senescence, or apoptosis in order to prevent tumor progression [10,11]. In unstressed cells, p53 is a short-lived protein that is maintained at very low levels by proteasome degradation. In response to various stresses, p53 is stabilized through multiple posttranslational modifications such as phosphorylation, acetylation, and methylation [10]. The acetylation of p53 has been shown to enhance its transactivation abilities and stability. p53 acetylation also enhances its sequence-specific DNA-binding activity. p53-mediated growth arrest and apoptosis were completely abrogated in mice with a lysine-to-arginine mutation at the major acetylation sites of p53 [12]. p53 acetylation is catalyzed by histone acetyltransferases including p300, cAMP response element binding protein-binding protein (CBP), p300/CBP-associated factor, Tat-interactive protein of 60 kDa (TIP60), and males absent on the first (MOF) [13].

Acetylated p53 is deacetylated by multiple histone deacetylases (HDACs), including HDAC1/2 and SIRT1 [10]. Various oncogenes have been shown to inhibit p53 acetylation, resulting in the inhibition of p53 functions. Mdm2 and TRB1 have been shown to induce p53 deacetylation by recruiting HDAC1 to p53 [14,15]. Oncoprotein Ski interacts with SIRT1, which promotes complex formation between p53 and SIRT1, leading to the deacetylation of p53 [16]. Shi et al. also showed that DEAD (Asp-Glu-Ala-Asp) box RNA helicase 24 inhibited p300-dependent p53 acetylation by blocking the p300-p53 interaction [17]. Thus, many oncogenes inactivate the tumor suppressor activities of p53 by inducing p53 deacetylation via various mechanisms.

Accumulating evidence suggests a complex and fine-tuning regulatory network connecting the p53 and Hippo pathways in a cellular context-dependent manner [18]. Another ortholog of Yorkie, Yes-associated protein (YAP), was shown to interact with and enhance p73-dependent apoptosis in response to DNA damage [19]. In contrast, a p53 mutant cooperated with YAP and TAZ to promote tumorigenesis [20]. Importantly, TAZ is required for self-renewal and tumor initiation abilities in breast cancer stem cells (CSCs) [18,21], while p53 functions as a barrier to the formation of CSCs [22]. However, physiological crosstalk between wild-type (WT) p53 and TAZ has not yet been clarified. We herein demonstrated that TAZ is a negative regulator of p53. The overexpression of TAZ antagonized p53 transcriptional activity, whereas its knockdown enhanced p53 transcriptional activity and decreased cell proliferation. As an underlying mechanism of action, TAZ suppressed the p300-mediated acetylation of p53 and reduced p53 DNA-binding activity. Moreover, TAZ knockdown induced p53-dependent cellular senescence in normal human fibroblasts. These results suggest that TAZ is a negative regulator of endogenous p53, and may contribute to tumorigenesis by suppressing p53-mediated cellular senescence.

## 2. Materials and Methods

### 2.1. Cell Culture and Transfection

H1299 (p53-null) cells were cultured in RPMI1640 medium (Sigma, St. Louis, MO, USA) supplemented with 10% (*v*/*v*) fetal bovine serum (FBS) (Sigma), penicillin G (100 units/mL) (Meiji Seika Pharma, Tokyo, Japan), and streptomycin (100 μg/mL) (Meiji Seika Pharma) at 37 °C in a 5% CO_2_ incubator [23]. MCF7 (wild-type p53) cells, HCT116 (wild-type p53) cells, and TIG-1 (wild-type p53) cells were cultured in Dulbecco’s modified Eagle’s medium (DMEM) (Sigma) supplemented with 10% FBS, penicillin G (100 units/mL), and streptomycin (100 μg/mL) at 37 °C in a 5% CO_2_ incubator [24].

Regarding DNA transfection, plasmids were transiently transfected with polyethylenimine (PEI) (Polysciences, Warrington, PA, USA) or Lipofectamine 2000 (Invitrogen, Carlsbad, CA, USA). In short interfering RNA (siRNA) transfection, siRNAs were transfected using Lipofectamine RNAiMAX (Invitrogen). Human *TAZ* siRNA (sense: 5′-AGACAUGAGAUCCAUCACUAA-3′) was purchased from FASMAC (Kanagawa, Japan). siRNA oligo targeting human *p53* mRNA was previously described [25]. Stealth RNAi^TM^ siRNA Luciferase Reporter Control (Invitrogen) was used as a control.

### 2.2. Plasmids

The original constructs encoding human p53, p300, SIRT1 and β-galactosidase (β-gal) were described previously [16,25]. p53RE-Luc (pGL4/p53RE) and *p21* promoter-Luc (pGL4/p21) have been described previously [23,25]. *NOXA* promoter-Luc (−198 to +45) was generated by ligating the human *NOXA* promoter region [26] with pGL4.10. pSUPERretro-p53 was described previously [27]. The *TAZ* Mission shRNA plasmid (TRCN0000319150) was obtained from Sigma. cDNA encoding TAZ was amplified by PCR and cloned into FLAG-pcDNA3, HA-pcDNA3, 6Myc-pcDNA3, or pGEX6P1 (GE Healthcare, Chicago, IL, USA). YAP was amplified by PCR and cloned into FLAG-pcDNA3. The tetracycline-inducible lentiviral pCW57.1-FLAG-p53 vector was generated by subcloning FLAG-p53 from pcDNA3-FLAG-p53 [16] into pCW57.1. pCW57.1 was a gift from David Root (Addgene plasmid #41393). All constructs were confirmed by DNA sequencing.

### 2.3. Antibodies and Reagents

An anti-p53 antibody (sc-126), horseradish peroxidase (HRP)-conjugated anti-p53 antibody (SC-126 HRP), anti-p21 antibody (sc-6246), anti-GST antibody (sc-138), and HRP-conjugated anti-HA antibody (SC-7392 HRP) were purchased from Santa Cruz Biotechnology (Santa Cruz, CA, USA). An anti-phospho-p53 (Ser15) antibody (9284), anti-acetyl-p53 (Lys382) antibody (2525), anti-PARP antibody (9542), and anti-TAZ antibody (4883) were purchased from Cell Signaling Technology (Beverly, MA, USA). An anti-Mdm2 antibody (OP46) was purchased from Calbiochem (San Diego, CA, USA). An anti-FLAG (M2) antibody (F3165), anti-β-actin antibody (A5441), actinomycin D (A9415), and Nutlin-3 (SML0580) were purchased from Sigma. An anti-phospho-p53 (Ser46) antibody (71-115) was obtained from BioAcademia (Osaka, Japan).

### 2.4. Luciferase Assay

H1299 cells were transfected with the luciferase reporter plasmid, expression plasmids, pCMV/β-gal, and an empty vector. The total amount of transfected DNA was the same in each experiment. Twenty-four hours after transfection, cells were harvested and luciferase activity was measured. Luciferase activity was normalized against β-gal activity [25].

### 2.5. Immunoprecipitation and Immunoblotting

Cells were lysed in TNTE buffer (20 mM Tris-HCl, pH 7.5, 120 mM NaCl, 1 mM EDTA, and 0.5% Triton X 100) supplemented with protease inhibitors and phosphatase inhibitors [28]. After being incubated for 15 min on ice, lysates were centrifuged at 15,000 rpm at 4 °C for 5 min and the supernatants were mixed with SDS sample buffer. Samples were then boiled for 5 min and resolved by SDS-PAGE. In anti-FLAG immunoprecipitation, cell lysates were incubated with anti-FLAG monoclonal antibody-conjugated M2 agarose beads (Sigma) at 4 °C for 2 h and washed four times with lysis buffer. Immunoprecipitated products were eluted with 3× FLAG peptide (Sigma) on ice for 30 min, and resolved by SDS-PAGE. Endogenous TAZ proteins were immunoprecipitated with an anti-TAZ antibody that had been pre-incubated for 6 h with Dynabeads Protein A (Invitrogen). Immunoprecipitates were washed five times with lysis buffer and then subjected to SDS-PAGE. After electrophoresis, the proteins were transferred to polyvinylidine difluoride membranes (Millipore, Bedford, MA, USA) and probed with the indicated antibodies. Immunoreactive proteins were visualized using enhanced chemiluminescence immunoblotting detection reagents (GE Healthcare), and light emission intensity was quantified with the Lumino-image analyzer LAS-3000 mini (GE Healthcare).

### 2.6. GST Pull-Down Assay

The recombinant p53 protein fused with GST was produced in *Escherichia coli*, and GST fusion proteins were purified as recommended by the instructions provided (GE Healthcare). H1299 cells were transfected with tagged-TAZ and TAZ deletion mutants. After 24 h, cells were lysed in TNTE buffer supplemented with protease inhibitors. Cell lysates were incubated with GST or GST-p53 at 4 °C for 2 h. Bound proteins were analyzed by immunoblotting.

### 2.7. RNA Isolation and Quantitative PCR (qPCR)

Total RNA from cells was extracted using RNAiso Plus (Takara Bio Inc., Shiga, Japan) and reverse-transcribed with the PrimeScript first-strand cDNA Synthesis Kit (Takara Bio Inc.) following the manufacturer’s instructions. Quantitative PCR was performed using SYBR premix Ex Taq (Takara Bio Inc.) and the ABI Prism 7300 sequence detection system (Applied Biosystems, Foster City, CA) with the following primers: 5′-GATTTCTACCACTCCAAACGCC-3′ and 5′-AGAAGATGTAGAGCGGGC-3′ for *p21* expression; 5′-TGTTGGTGCACAAAAAGACA-3′ and 5′-CACGCCAAACAAATCTCCTA-3′ for *Mdm2* expression; 5′-GGCTGGGAGATGACCTTCAC-3′ and 5′-CTGAGTGGGGTGGTTCTGCT-3′ for *TAZ* expression; 5′-TTTCACCCTTCAGATCCGTGG-3′ and 5′-TTCCAAGGCCTCATTCAGCTC-3′ for *p53* expression; 5′-TTTGCTTTCCTTGGTCAGGC-3′ and 5′-GCTTGCGACCTTGACCATCT-3′ for *HPRT1* expression; 5′-TGGCACCCAGCACAATGAA-3′ and 5′-CTAAGTCATAGTCCGCCTAGAAGCA-3′ for *β-actin* expression.

### 2.8. Chromatin Immunoprecipitation (ChIP) Assay

Cells were crosslinked with 1% formaldehyde, and then lysed in SDS lysis buffer (50 mM Tris-HCl, pH 8.0, 1% SDS, 10 mM EDTA, and protease inhibitors). The ChIP procedure was performed as previously described [15]. The primers used in the present study were 5′-GTGGCTCTGATTGGCTTTCTG-3′ and 5′-CTGAAAACAGGCAGCCCAAG-3′ for the *p21* promoter (-2283), 5′-CCAGGAAGGGCGAGGAAA-3′ and 5′-ACATCTCAGGCTGCTCAGAGTCT-3′ for the p21 promoter (-1391), 5′-TGTTTGGGCTATTTACTAGTTG-3′, and 5′-ATAAAATGACTTAAGCCCAGAG-3′ for the *HPRT1* first intron primers.

### 2.9. Cell Viability Assay, Apoptosis Assay, and Senescence-Associated β-gal (SA-β-gal) Staining

Cells were washed with PBS. Crystal violet solution was added to stain cells, and stained cells were dissolved in 1% SDS and absorbance was measured at 595 nm. For apoptosis assay, both floating and attached cells were collected 48 h after Nutlin-3 treatment. Cells were then stained with Annexin V-FITC using the FITC Annexin V Apoptosis Detection Kit (BD Biosciences, Franklin Lakes, NJ, USA) [23]. SA-β-gal staining was performed as previously described [29].

### 2.10. Statistical Analysis

The significance of differences between two groups was evaluated using two-tailed Student’s *t*-test. In multi-group analyses, significance was assessed using a one-way ANOVA with the post hoc Tukey-Kramer HSD test.

## 3. Results

### 3.1. TAZ Represses the Transcriptional Activity of p53

**Figure 1:**
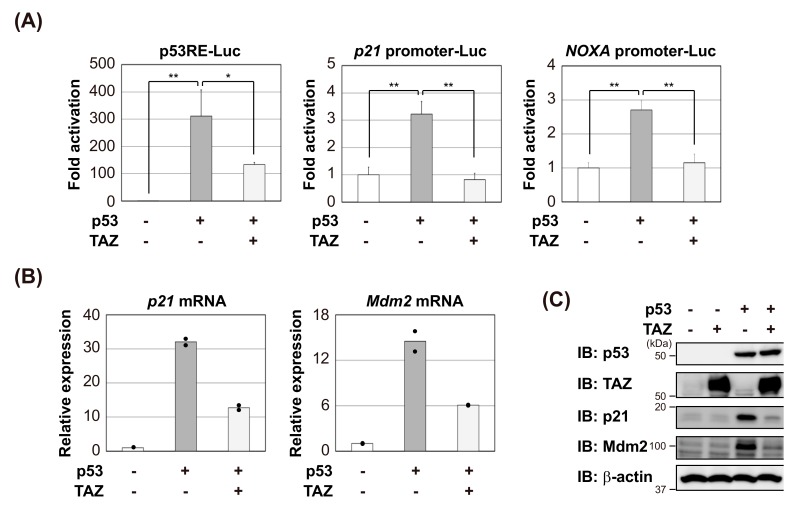
TAZ represses the transcriptional activity of p53. (**A**) H1299 cells were transfected with the indicated reporter plasmids and pCMV/β-gal in combination with the indicated constructs. After 24 h, luciferase activity in cell lysates was measured and normalized with β-gal activity. Experiments were performed in triplicate, and data are represented as mean activation fold ± S.D. (**B**,**C**) H1299 cells were transfected with p53 in the absence or presence of TAZ. After 18 h, p53 target genes expression levels were analyzed by RT-qPCR (duplicate determination) (**B**) or immunoblotting (**C**). Significant differences are indicated as ** *p* < 0.01 and * *p* < 0.05.

Since TAZ is known to promote the development and progression of cancer, we investigated whether it affects the transcriptional activity of the tumor suppressor p53. p53-null H1299 cells were co-transfected with p53 and the p53-specific luciferase reporter, p53RE-Luc, in the absence or presence of TAZ. The transcriptional ability of p53 was repressed by the co-expression of TAZ (Figure 1A, left). Similar results were obtained in experiments using *p21* promoter-Luc (Figure 1A, middle) or *NOXA* promoter-Luc (Figure 1A, right). As a side note, YAP also repressed the transcriptional activity of p53 (Supplementary Figure S1). To confirm the effects of TAZ on p53 transcriptional activity, the induction of endogenous p53 target genes was monitored by reverse transcriptase-coupled quantitative PCR (RT-qPCR) and immunoblotting. As shown in Figure 1B,C, the exogenous expression of TAZ suppressed the expression levels of p21 and Mdm2.

**Figure 2:**
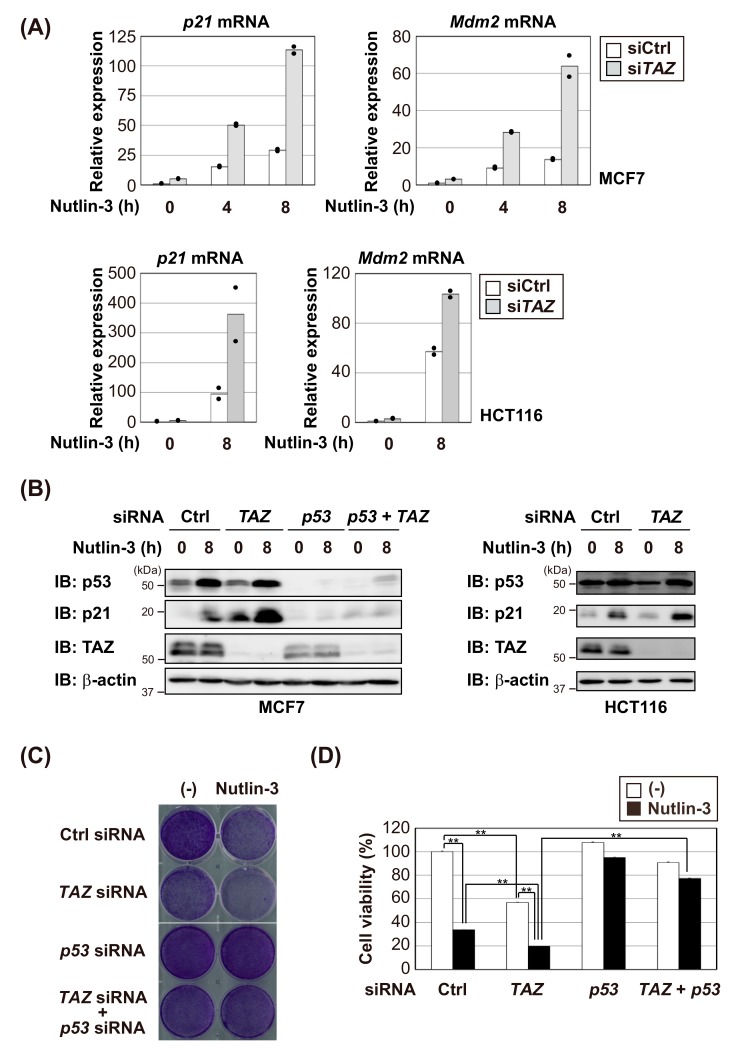
TAZ knockdown enhances the transcriptional activity of p53. (**A**,**B**) MCF7 cells and HCT116 cells were transfected with the indicated siRNAs and treated with 10 μM Nutlin-3 for 8 h. The expression levels of p53 target genes were analyzed by RT-qPCR (duplicate determination) (A) or immunoblotting (**B**). (**C**,**D**) TAZ knockdown reduced cell viability after the Nutlin-3 treatment. MCF7 cells were transfected with the indicated siRNAs, and then treated with 10 μM Nutlin-3. After 48 h, cells were stained with crystal violet (**C**). The quantification of data is represented (**D**). Significant differences are indicated as ** *p* < 0.01.

We then investigated whether TAZ knockdown increases the transcriptional activity of p53. Therefore, siRNA was used to knockdown TAZ in MCF7 and HCT116 cells. *TAZ* siRNA increased *p21* and *Mdm2* mRNA levels and p21 protein expression induced by Nutlin-3, a non-genotoxic activator of the p53 pathway [30], more than control siRNA in both cell lines (Figure 2A,B). Furthermore, the simultaneous knockdown of p53 and TAZ in MCF7 cells canceled this Nutlin-3-induced increase in p21 expression (Figure 2B). Under high cellular density conditions, TAZ is phosphorylated and inactivated by the Hippo pathway activation [3,4]. We found that high density cell culture enhanced the p21 induction upon the actinomycin D (Act D) treatment, which activated the ribosomal stress response to stabilize the p53 protein [31], suggesting that the transcriptional activity of p53 is regulated by the Hippo pathway through TAZ (Supplementary Figure S2). We then investigated the physiological effects of the negative regulation of p53 by TAZ. As expected, the knockdown of TAZ in MCF7 cells reduced cell viability due to the up-regulation of p53 target genes (Figure 2C,D). This effect decreased with the simultaneous knockdown of p53. Furthermore, the knockdown of TAZ in MCF7 cells caused a significant reduction in viable cells with Nutlin-3. Annexin V staining revealed that TAZ knockdown enhanced apoptotic cell death in MCF7 cells after exposure to Nutlin-3 (Supplementary Figure S3). These results indicate that TAZ suppresses p53 transactivation ability.

### 3.2. TAZ Interacts with p53

**Figure 3:**
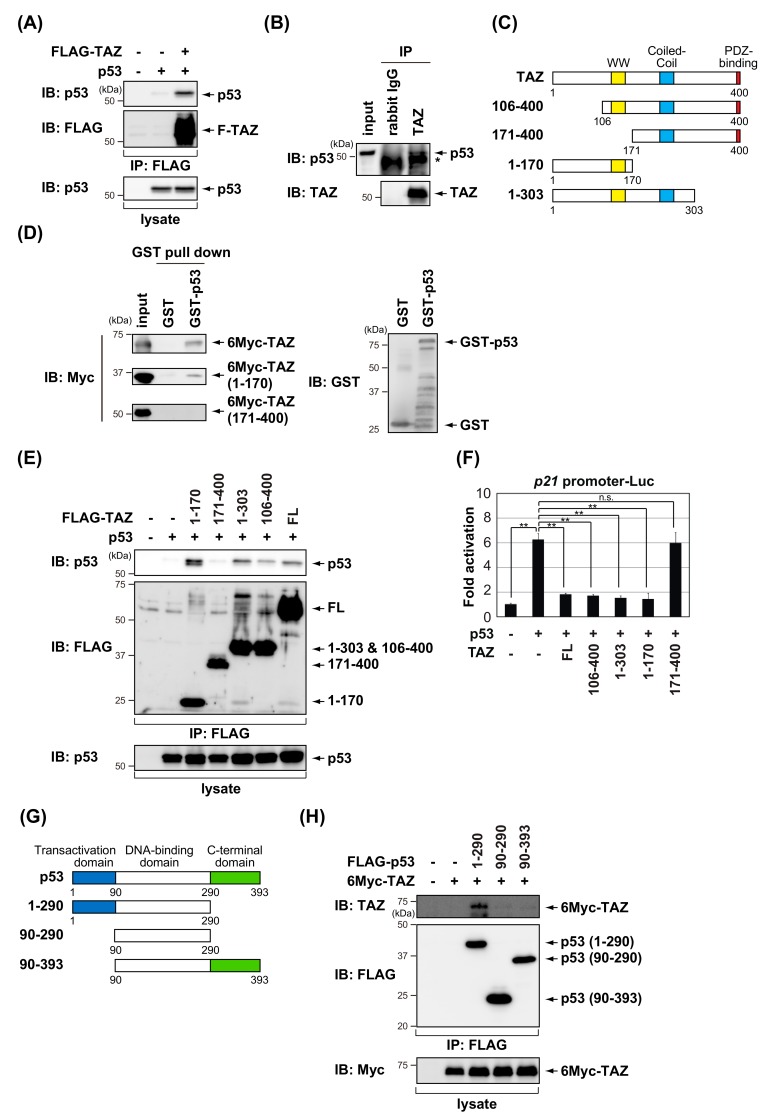
TAZ interacts with p53. (**A**) H1299 cells were transiently transfected with the indicated constructs. After 24 h, cell lysates were immunoprecipitated (IP) with an anti-FLAG antibody and then immunoblotted with the indicated antibodies. (**B**) MCF7 cells were treated with 10 μM MG132, a proteasome inhibitor, for 8 h. Cell lysates were immunoprecipitated with an anti-TAZ antibody and then immunoblotted with the indicated antibodies. The asterisk indicates heavy chain of immunoglobulin. (**C**) Schematic representation of full-length TAZ and its deletion mutants. (**D**) In vitro interaction of GST–p53 with TAZ. Twenty-four hours before harvesting, H1299 cells were transfected with 6Myc-TAZ or its deletion mutants. Cell lysates were subjected to GST pull down and then immunoblotted with the indicated antibodies. (**E**) H1299 cells were transfected with the indicated constructs. After 24 h, cell lysates were immunoprecipitated (IP) with the anti-FLAG antibody and then immunoblotted with the indicated antibodies. (**F**) H1299 cells were transfected with *p21* promoter-Luc and pCMV/β-gal in combination with the indicated constructs. After 24 h, luciferase activity in cell lysates was measured and normalized with β-gal activity. Experiments were performed in triplicate, and data are represented as mean activation fold ± S.D. (**G**) Schematic representation of full-length p53 and its deletion mutants. (**H**) H1299 cells were transfected with the indicated constructs. After 24 h, cell lysates were immunoprecipitated (IP) with the anti-FLAG antibody and then immunoblotted with the indicated antibodies. Significant differences are indicated as ** *p* < 0.01. n.s.: not significant.

Since TAZ suppresses p53-mediated transactivation, we investigated whether TAZ physically interacts with p53. FLAG-TAZ and p53 were transiently transfected into H1299 cells, and a co-immunoprecipitation analysis showed that p53 co-precipitated with TAZ (Figure 3A). The interaction between TAZ and p53 was also detected in the endogenous proteins of MCF7 cells (Figure 3B). A GST pull-down analysis was performed to map the TAZ domain to which p53 binds. Full-length and the N-terminal portion of TAZ (amino acids (a.a.) 1–170), not the C-terminal portion (a.a. 171–400), were able to bind GST–p53 (Figure 3C,D). We also examined the interaction between p53 and various FLAG-tagged TAZ deletion mutants (Figure 3C,E). The deletion of residues 1–105 (a.a. 106–400) had no effect on binding to p53, whereas the further truncation of TAZ to residue 170 (a.a. 171–400) weakened the p53 interaction. In addition, a reporter assay using the *p21* promoter showed that p53-dependent transactivation was not inhibited by the expression of the C-terminal portion of TAZ (Figure 3F). These results suggest that the region of residues 106–170 of TAZ containing the WW domain is required for p53 binding. To map the TAZ-binding domain in p53, we examined the interaction between TAZ and various p53 deletion mutants (Figure 3G) using a co-immunoprecipitation analysis. As shown in Figure 3H, TAZ bound to p53 (a.a. 1–290), but not to p53 (a.a. 90–290) or p53 (a.a. 90–393). These results suggest that the p53 transactivation domain is required for the interaction with TAZ.

### 3.3. TAZ Suppresses p300-Mediated Acetylation of p53 and Reduces the DNA-Binding Activity of p53

**Figure 4:**
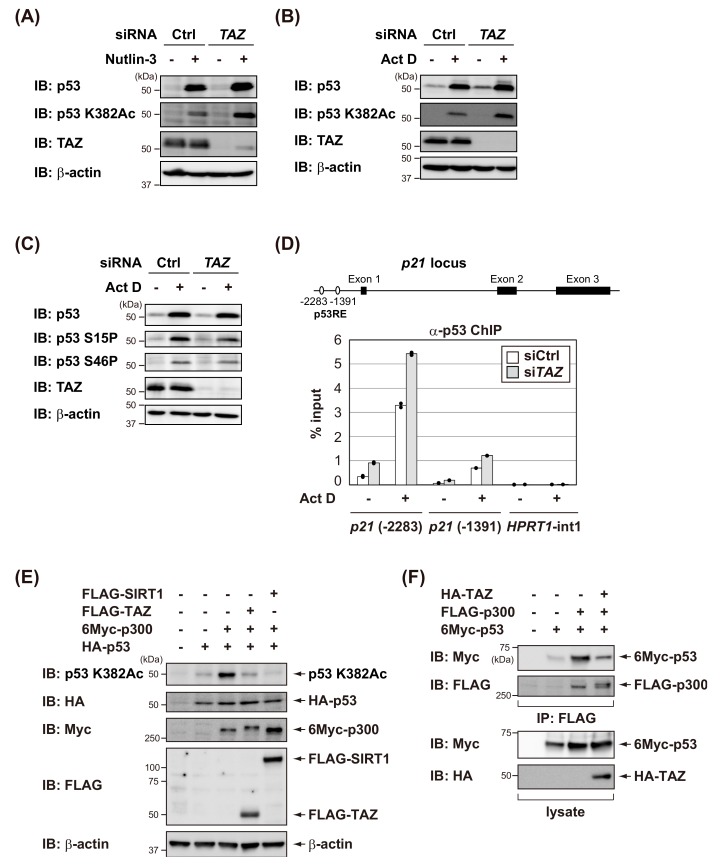
TAZ suppresses p53 acetylation by p300 and reduces the DNA-binding activity of p53. (**A**) MCF7 cells were transfected with control (Ctrl) or *TAZ* siRNA, and treated with 10 μM of Nutlin-3 for 8 h. Cell lysates were analyzed by immunoblotting using the indicated antibodies. (**B**,**C**) MCF7 cells were transfected with control (Ctrl) or *TAZ* siRNA, and treated with 5 nM of actinomycin D (Act D) for 8 h. Cell lysates were analyzed by immunoblotting using the indicated antibodies. (**D**) Schematic of the *p21* locus indicating the two p53 responsive elements (p53REs) (upper). MCF7 cells were transfected with the indicated siRNAs for 48 h and then treated with 5 nM of ActD for 4 h. Chromatin immunoprecipitation (ChIP) was performed using an anti-p53 antibody, and qPCR (duplicate determination) was performed for the indicated promoters (lower). (**E**) H1299 cells were transfected with the indicated constructs, and the level of acetylated p53 was assessed by immunoblotting using an anti-acetylated p53 antibody (p53 K382Ac). (**F**) H1299 cells were transfected with the indicated constructs, and FLAG-p300 was immunoprecipitated (IP) with anti-FLAG antibodies. Co-precipitated 6Myc-p53 was detected by immunoblotting using the anti-Myc antibody.

p53 transcriptional activation is normally regulated by posttranslational modifications, such as phosphorylation and acetylation, in response to various stresses [32]. We investigated whether TAZ knockdown affected p53 phosphorylation and acetylation. As shown in Figure 4A,B, the knockdown of TAZ increased acetylation levels of p53 after Nutlin-3 or Act D treatments in MCF7 cells. On the other hand, TAZ knockdown in MCF7 cells did not significantly affect p53 phosphorylation after Act D treatment (Figure 4C). These results suggest that TAZ suppressed acetylated p53 levels in response to stresses.

Since p53 acetylation increases the abilities of p53 to bind to DNA, the ChIP assay was used to examine the DNA-binding activity of p53 to the *p21* promoter. The *p21* promoter has two p53 responsive elements (p53REs), the distal site at −2283 that binds p53 relatively strongly and the proximal site at −1391 that is more weakly bound by p53 [33]. The Act D treatment increased the ability of p53 to bind to the *p21* promoter, but not to the region used as the control (*HPRT1* first intron). However, as expected, TAZ knockdown increased the amount of p53 bound to DNA (Figure 4D). Thus, TAZ appears to suppress p53 DNA binding by inhibiting p53 acetylation, thereby inactivating p53.

p300 functions as a transcriptional coactivator and is responsible for acetylating p53 [34]. Therefore, we investigated whether TAZ antagonizes p300-mediated p53 acetylation. As shown in Figure 4E, p300 promoted p53 acetylation in H1299 cells, whereas the deacetylase SIRT1 inhibited p300-mediated p53 acetylation. Similarly, TAZ expression antagonized p300-mediated p53 acetylation in H1299 cells. Furthermore, TAZ expression attenuated the interaction between p53 and p300 (Figure 4F). These results suggest that TAZ suppresses p53-p300 binding, thereby inhibiting p300-mediated p53 acetylation.

### 3.4. TAZ Knockdown Induces p53-Dependent Senescence in Normal Human Fibroblasts

**Figure 5:**
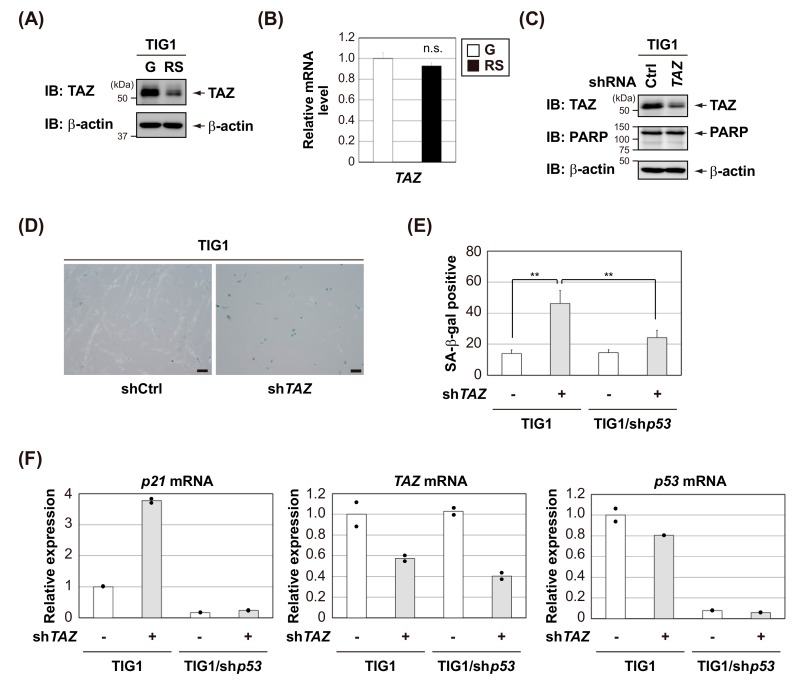
TAZ knockdown induces p53-dependent senescence in normal human fibroblasts. (**A**) TAZ was down-regulated in replicative senescent TIG1 cells. Total protein was extracted from TIG1 cells with PD30 (G: growing) or PD65 (RS: replicative senescence), and immunoblotting analysis was performed with the indicated antibodies. (**B**) Total RNA was extracted from TIG1 cells with PD30 (G) or PD65 (RS), and the expression of *TAZ* mRNA was assessed by qPCR. mRNA level of *TAZ* was normalized with *β-actin* mRNA. Result is shown as means ± S.D (*n* = 3). (**C**–**F**) TIG1 cells were infected with lentiviral vectors containing shRNA for *TAZ* (sh*TAZ*) or control (shCtrl) for 7 d. Total cell lysates were harvested for an immunoblot analysis with the indicated antibodies (**C**). Cells were then stained for senescence-associated β-gal (SA-β-gal). Representative images of the indicated cells stained for SA-β-gal activity (scale bar, 100 μm) (**D**). The bar graph shows the percentage of SA-β-gal–positive cells in the indicated culture (**E**). The expression of each gene was assessed by qPCR (duplicate determination), and the mRNA levels of the indicated genes were normalized with *β-actin* mRNA (**F**). Significant differences are indicated as ** *p* < 0.01. n.s.: not significant.

Cellular senescence is one of the tumor suppressor mechanisms that prevents the proliferation of pre-malignant cells [35]. The senescence program is initiated and maintained by the p53-p21 and p16^Ink4a^-pRB pathways [36]. In the present study, we found that TAZ negatively regulated the transcriptional activity of p53; therefore, further studies are needed to clarify whether the deletion of TAZ affects p53-dependent cellular senescence. At first, we assessed the expression levels of TAZ in normal human diploid fibroblast TIG1 cells triggered to undergo replicative senescence. Interestingly, we found that TAZ protein levels decreased, whereas *TAZ* mRNA levels did not vary in the late passage of TIG1 cells (Figure 5A,B). These results suggest that the reduction in TAZ protein levels associated with replicative senescence may be regulated at the post-translational level. To investigate whether TAZ affects cellular senescence, lentiviral shRNA was used to knockdown TAZ in TIG1 cells. As shown in Figure 5C–E, TAZ knockdown induced senescence, not apoptosis, in TIG1 cells. TAZ knockdown increased *p21* mRNA levels in TIG1 cells (Figure 5F). Importantly, the further depletion of p53 suppressed TAZ knockdown-induced senescence and *p21* mRNA up-regulation in TIG1 cells (Figure 5E,F), but these effects were largely rescued by p53 reconstitution (Supplementary Figure S4). Collectively, these results suggest that TAZ regulates cellular senescence by negatively regulating p53 transcriptional activity.

## 4. Discussion

**Figure 6:**
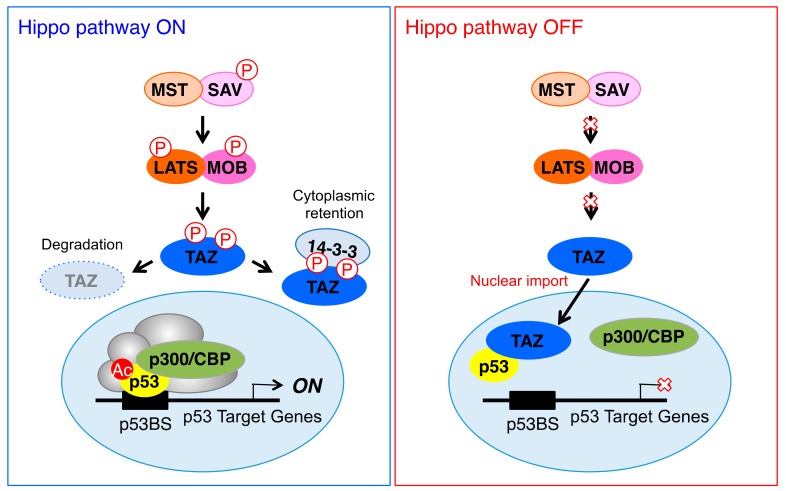
A hypothetical model of p53 transcriptional activity control by TAZ. When the Hippo pathway is activated, TAZ is phosphorylated by its upstream kinases, MST1/2 and LATS1/2. As a result, TAZ is either degraded by the ubiquitin-proteasome system or bound to 14-3-3 and sequestered in the cytoplasm (**left**). However, mutations in or the altered expression of MST1/2 and LATS1/2 lead to increased TAZ nuclear localization and activation in cancer cells. Nuclear TAZ suppresses binding between p53 and p300, thereby inhibiting the p300-mediated acetylation of p53. Consequently, TAZ negatively regulates p53 functions by inhibiting p53 transcriptional activity and positively enhances cell proliferation (**right**).

In the present study, we demonstrated that TAZ negatively regulated p53 functions by inhibiting the transcriptional activity of p53 and positively controlled cell proliferation (Figure 6). In response to various stresses, p53 suppresses tumorigenesis by initiating cell functions, such as cell cycle arrest or apoptosis. p53 also functions as a barrier to prevent the acquisition of the stemness properties of CSCs. On the other hand, TAZ has been reported to play an important role in maintaining the function of CSCs. Therefore, p53 and TAZ antagonize their functions during cancer development and progression. Inactivation of the p53 pathway is closely associated with cancer development, with approximately 50% of human cancers showing mutations in the *TP53* gene.

In addition, the inactivation of p53 functions by p53-associated proteins has been reported in cancers carrying wild-type p53. Therefore, based on the present results, TAZ has potential as a molecular target for cancers carrying wild-type p53.

The present results also suggest that the region containing the WW domain of TAZ is important for the regulation of p53 transcriptional activity (Figure 3E,F). The WW domain is critical for regulating TAZ function, which is known to be to bind to transcriptional factors, such as TEA domain family members, T-box transcription factor 5 (TBX5), and Runt-related transcription factor 2 (Runx2) [37]. The peptidylprolyl cis/trans isomerase, NIMA-interacting 1 (Pin1) is a protein that contains the WW domain [38]. The WW domain of Pin1 binds to the phosphorylated serine/threonine-proline (pS/T-P) motif [39]. The binding region with TAZ in p53 is the N-terminal transcriptional activation domain, which contains pS/T-P motifs (Figure 3H). Therefore, the p53-TAZ interaction may be regulated by the phosphorylation of the N-terminal domain of p53 upon cellular stress. Further studies are warranted.

TAZ and YAP have distinct and overlapping functions [40]. YAP knockout embryos die at embryonic day 8.5 due to a severe developmental disorder [41]. In contrast, TAZ knockout mice survive only up to 3 weeks of age, but are viable, characterized by renal cysts, and exhibit end-stage renal disease [42]. Jeong et al. recently reported that a TAZ deficiency increased the level of apoptosis and senescence in spermatogenic cells and Leydig cells [43]. They also showed that TAZ may suppress spermatogenic cell apoptosis and senescence by inhibiting p53 activity, consistent with the present results. Santinon et al. also demonstrated that YAP and TAZ avoided oncogene-induced cellular senescence [44]. In the present study, we found that TAZ knockdown increased p21 expression and induced cellular senescence in normal fibroblast TIG1 cells (Figure 5). TAZ knockdown-induced cell senescence was suppressed by the further knockdown of p53, suggesting the existence of an inhibitory effect of TAZ in p53-mediated cell senescence induction. However, since TAZ regulates the expression of various proliferative and anti-apoptotic genes, it may be involved in senescence independent of p53.

TAZ is also involved in epithelial–mesenchymal transition (EMT), which contributes to cancer invasion and metastasis [45]. We herein reported that TAZ contributes to cancer progression by suppressing p53 functions. Since p53 has been reported to function as a barrier to the formation of CSCs [22], TAZ is considered to influence the effects of p53, which inhibits CSC functions. Based on these findings, the development of molecular targeted drugs that suppress TAZ activity is considered promising for advances in cancer treatment. The biological activity of TAZ is essential for embryonic tissue growth, whereas this activity does not appear to be essential in the homeostasis of adult tissue. Although the impact of TAZ knockout on the phenotypes of kidney and lung maturation in mice needs to be considered, the development of anticancer drugs targeting TAZ may be effective in treating cancers that strongly express TAZ carrying wild-type p53. Statins, which are 3-hydroxy-3-methylglutaryl coenzyme A (HMG-CoA) reductase inhibitors, are candidates for suppressing TAZ activity. A previous study reported that geranylgeranyl pyrophosphate produced by the mevalonate pathway activated Rho small GTPases, which, in turn, activated YAP/TAZ by reducing phosphorylation and caused nuclear localization [46]. One of the anticancer effects of statins is the suppression of cell proliferation by inducing p21 [47]. Notably, several population-based retrospective studies showed statin chemoprevention and survival benefits in various types of cancers [48]. We consider the present results to support the potential of TAZ as a diagnostic marker and the rationale for targeting TAZ as a potential tumor treatment.

## Supplementary Materials

The following are available online at https://www.mdpi.com/2073-4409/9/1/171/s1, Figure S1: YAP represses the transcriptional activity of p53. Figure S2: High cellular density enhanced p21 induction in MCF7 cells after Act D treatment. Figure S3: TAZ knockdown enhanced apoptotic cell death in MCF7 cells after Nutlin-3 treatment. Figure S4: p53 reconstitution largely restored cellular senescence and *p21* induction by TAZ knockdown in p53 knockdown TIG1 cells.
