# Supplementary material for: Transcriptional Coactivator TAZ Negatively Regulates Tumor Suppressor p53 Activity and Cellular Senescence
Source: Cells. 2020 Jan 9;9(1):171. doi: 10.3390/cells9010171 (PMC7016652; doi:10.3390/cells9010171)
Supplement: Supplementary file 1 [file cells-09-00171-s001.pdf]

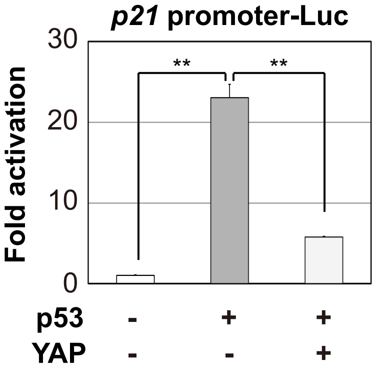

Supplementary Figure S1. YAP represses the transcriptional activity of p53. H1299 cells were transfected with a p53 reporter plasmid, *p21*-promoter Luc and pCMV/β-gal in combination with the indicated constructs. After 24 h, luciferase activity in cell lysates was measured and normalized with β-gal activity. Experiments were performed in triplicate, and data are represented as mean activation fold ± S.D. Significant differences are indicated as **$p<0.01$.

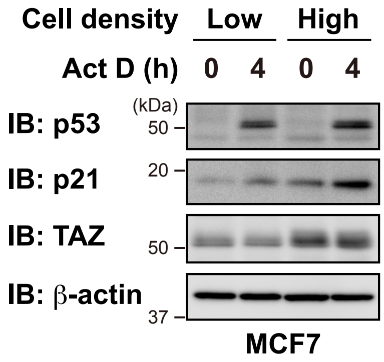

Supplementary Figure S2. MCF7 cells were cultured at low density (0.5 x 10^5 cells/well) or high denisty (1.0 x 10^6 cells/well). After 24 h, cells were treated with 10 nM actinomycin D (Act D) for 4 h. Cell lysates were analyzed by immunoblotting using the indicated antibodies.

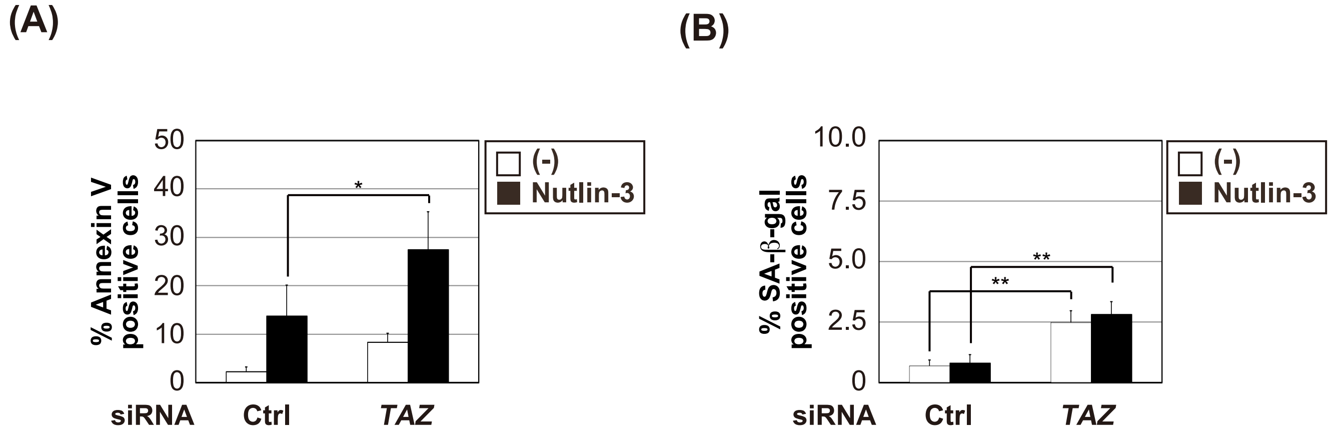

Supplementary Figure S3. MCF7 cells were transfected with the indicated siRNAs. After 48 h, cells were treated with 10 μM Nutlin-3 for 48 h. Quantitation of apoptotic cell death was examined by Annexin V staining. Shown are average results from three experiments (A). Cells were then stained for senescence-associated β-gal (SA-β-gal). The bar graph shows the percentage of SA-β-gal–positive cells in the indicated culture (n=3) (B).
Significant differences are indicated as **$p<0.01$ and *$p<0.05$.

(A)

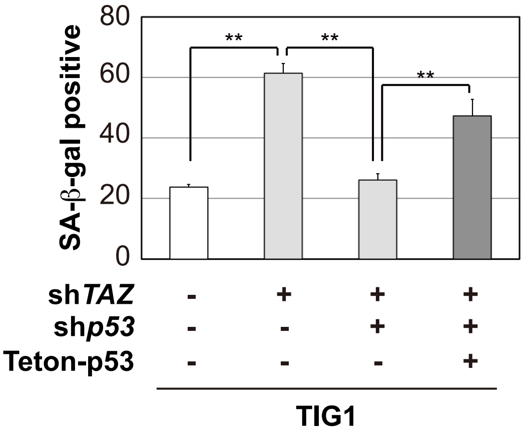

(B)

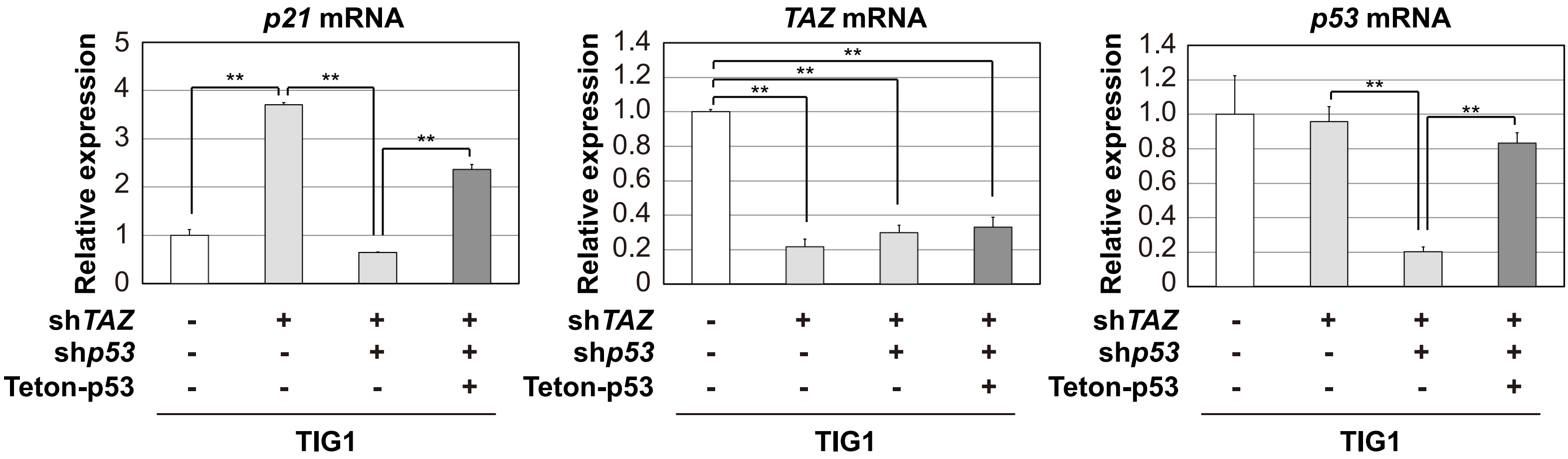

Supplementary Figure S4. TIG1 cells or TIG1/shp53 cells expressing Tetracycline-inducible p53 (Teton-p53) were infected with lentiviral vectors containing shRNA for *TAZ* (sh*TAZ*) or control (shCtrl) in the presence (Teton-p53) or absence of doxycycline (1 μg/ml) for 7 d. Cells were then stained for senescence-associated β-gal (SA-β-gal). The bar graph shows the percentage of SA-β-gal–positive cells in the indicated culture (n=3) (A). The expression of each gene was assessed by qPCR, and the mRNA levels of the indicated genes were normalized with *β-actin* mRNA. Result was shown as means ± S.D. (n=3) (B). Significant differences are indicated as **p<0.01.
